# Oxidative stress biomarkers in Fabry disease: is there a room for them?

**DOI:** 10.1007/s00415-020-10044-w

**Published:** 2020-07-27

**Authors:** C. Simoncini, S. Torri, V. Montano, L. Chico, F. Gruosso, A. Tuttolomondo, A. Pinto, I. Simonetta, V. Cianci, A. Salviati, V. Vicenzi, G. Marchi, D. Girelli, D. Concolino, S. Sestito, M. Zedde, G. Siciliano, Michelangelo Mancuso

**Affiliations:** 1 Department of Clinical and Experimental Medicine, Neurological Institute, University of Pisa, Pisa, Italy; 2 Internal Medicine and Stroke Care Ward, Department of Promoting Health, Maternal-Infant, Excellence and Internal and Specialized Medicine (Promise) G. D’Alessandro, University of Palermo, Palermo, Italy; 3 Regional Epilepsy Centre, Great Metropolitan Hospital “Bianchi-Melacrino-Morelli”, Reggio Calabria, Italy; 4 Lab Functional Genomics, Department of Biotechnology, Univ Verona, Genartis srl, Verona, Italy; 5 Medical Genetics Unit, ASL 9, Verona, Italy; 6 Internal Medicine Unit, Azienda Ospedaliera Universitaria Integrata di Verona, Verona, Italy; 7 Department of Medical and Surgical Sciences, Pediatric Unit, “Magna Graecia” University, Catanzaro, Italy; 8 Azienda Unità Sanitaria Locale, IRCCS di Reggio Emilia, Reggio Emilia, Italy

**Keywords:** Fabry disease, Biomarkers, lysoGb3, Oxidative stress

## Abstract

**Background:**

Fabry disease (FD) is an X-linked lysosomal storage disorder, caused by deficient activity of the alpha-galactosidase A enzyme leading to progressive and multisystemic accumulation of globotriaosylceramide. Recent data point toward oxidative stress signalling which could play an important role in both pathophysiology and disease progression.

**Methods:**

We have examined oxidative stress biomarkers [Advanced Oxidation Protein Products (AOPP), Ferric Reducing Antioxidant Power (FRAP), thiolic groups] in blood samples from 60 patients and 77 healthy controls.

**Results:**

AOPP levels were higher in patients than in controls (*p* < 0.00001) and patients presented decreased levels of antioxidant defences (FRAP and thiols) with respect to controls (*p* < 0.00001). In a small group of eight treatment-naïve subjects with FD-related mutations, we found altered levels of oxidative stress parameters and incipient signs of organ damage despite normal lyso-Gb3 levels.

**Conclusions:**

Oxidative stress occurs in FD in both treated and naïve patients, highlighting the need of further research in oxidative stress-targeted therapies. Furthermore, we found that oxidative stress biomarkers may represent early markers of disease in treatment-naïve patients with a potential role in helping interpretation of FD-related mutations and time to treatment decision.

## Introduction

Fabry disease (FD) is an X-linked lysosomal storage disorder, caused by deficient activity of the alpha-galactosidase A enzyme (GAL-A), encoded by the *GLA* gene. The enzyme deficiency causes accumulation of the sphingolipid globotriaosylceramide (GL-3 or Gb3) and its deacylated derivative lyso-globotriaosylceramide (lyso-GL-3 or lyso-Gb3) in various cell types, in particular the vascular endothelial and the smooth muscle cells, cardiac myocytes, dorsal root ganglion neurons, neurons of the autonomic nervous system, brain and all types of kidney cells [1].

Although male patients are traditionally considered to develop a more severe phenotype than females, the disorder has a substantial clinical heterogeneity. The classical phenotype (type 1) exhibits early manifestations, whereas non-classical or late-onset phenotype (type 2) presents with clinical signs later in life [1]. In a multicentre study [2], classical FD was differentiated from non-classical FD by the presence of at least one of three clinical criteria (neuropathic pain, angiokeratoma, and cornea verticillata) and an additional biochemical criterion in males (leukocyte GAL-A activity < 5%). Although several mutations in *GLA* have been associated to classic or late-onset forms, a definite genotype–phenotype correlation is not demonstrated, and diagnosis and classification should not be made only on genetic-basis.

Substrates deposition is related to tissue damage in FD; however, the underlying molecular mechanisms remain not completely understood. Recent data highlighted the role of oxidative stress in FD pathophysiology. FD patients present high lipid and protein oxidative damage, decreased antioxidant defences and increased inflammatory biomarkers and cytokines [3–10]. Excess intracellular Gb3 induces oxidative stress and up-regulates the expression of cellular adhesion molecules in vascular endothelial cells [5]. Moreover, it has been suggested that pro-oxidant state occurs, is correlated and seems to be induced by Gb3 in Fabry patients [9].

### Hypothesis/study objective/purpose

The aim of our multicentre project was to evaluate the role of oxidative stress in FD. In particular, we have evaluated (i) if oxidative stress occurs in blood; (ii) if there is an association between oxidative stress biomarkers and FD clinical manifestations and\or a difference between classic and late onset FD; (iii) if oxidative stress parameters over time are related with Lyso-Gb3 and disease appearance or progression, in a subgroup of eight treatment-naïve subjects/patients with normal Lyso-Gb3 levels, in order to see if selected oxidative stress biomarkers could represent early markers of disease progression.

## Materials and methods

The patients group consisted of 60 Caucasian genetically proven FD subjects (Table 1) from Italy recruited from seven Italian centres with expertise in FD. Diagnosis and phenotype classification was performed according to international recommendations [1, 2], although few cases remained of uncertain interpretation as discussed below.

**Table 1:** demographic, molecular and clinical features of the FD patients enrolled in the study

	Gender	Mutation	Age of onset (years)	Clinical onset	Clinical involvement at last follow-up
1	F	c.337T > C p.Phe113Leu	33	–	–
2	F	c.337T > C p.Phe113Leu	27	–	–
3	M	c.337T > C p.Phe113Leu	62	Myocardial infarction	HCM, Arrhythmia, Myocardial infarction
4	M	deletion exons 3, 4	9	Acroparesthesia	Acroparesthesia, HCM
5	M	c.337T > C p.Phe113Leu	62	Arrhythmia	Arrhythmia, HCM, Proteinuria, VBD
6	F	deletion exons 3, 4	50	Acroparesthesia	Acroparesthesia
7	M	c.818T > C p.Phe273Ser	22	Acroparesthesia	Acroparesthesia, HCM, Arrhythmia, Proteinuria, Stroke, VBD
8	F	c.337 T > C p.Phe113Leu	40	Stroke	Stroke, Proteinuria
9	F	c.550T > A	39	HCM	HCM, Stroke
10	F	IVS2-76_80 del15; IVS 4-16 A > G; IVS 6-22 G > T	30	Acroparesthesia	Acroparesthesia, Stroke
11	F	.-10 G > T, IVS 6-22 C > T; IVS 6-51_54 del14, g.9151 C > T	32	Acroparesthesia	Acroparesthesia, Proteinuria, Stroke, GI disorders
12	M	.-10 C > T; IVS 2-76_80 del 5; IVS 4-16 A > G; IVS 6-22 C > T	4	GI disorders	GI disorders, Acroparesthesia
13	F	.- 30 G > A	30	Acroparesthesia	Acroparesthesia, Stroke, GI disorders
14	F	.-10 C > T; IVS 2-76_80 del 5; IVS 4-16 A > G; IVS 6-22 C > T	35	Stroke	Stroke, Acroparesthesia
15	F	c.547 G > A p.G183S	17	Acroparesthesia	Acroparesthesia, Proteinuria
16	M	c.614delC	29	HCM	HCM, Acroparesthesia
17	F	c.614delC	12	Acroparesthesia	Acroparesthesia, Stroke, HCM
18	F	.-10 C > T; IVS 2-76_80 del 5; IVS 4-16 A > G; IVS 6-22 C > T	30	Acroparesthesia	Acroparesthesia
19	M	.-10 C > T; IVS 2-76_80 del 5; IVS 4-16 A > G; IVS 6-22 C > T	10	GI disorders	GI disorders, Acroparesthesia, Proteinuria
20	F	c.547 G > A p.G183S	22	–	–
21	M	c.337 T > C p.Phe113Leu	40	Proteinuria	Proteinuria, HCM
22	F	c.1146C > A; p.C382X	12	Proteinuria	Proteinuria
23	F	p.Tyr222Asp	49	Proteinuria	Proteinuria, Arrhythmia, GI disorders
24	M	c.640-859C > T	32	HCM	HCM, Arrhythmia, Proteinuria, VBD, GI disorders
25	F	D313Y	31	VBD	VBD, GI disorders
26	F	p.Ala377Asp	32	Acroparesthesia	Acroparesthesia, Arrhythmia, Proteinuria
27	F	c.1146C > A; p.C382X	30	Acroparesthesia	Acroparesthesia, TIA, VBD, GI disorders
28	F	c.640-859C > T	52	Proteinuria	Proteinuria, HCM, Arrhythmia, VBD
29	F	p.Tyr222Asp	13	Acroparesthesia	Acroparesthesia, VBD, GI disorders
30	F	D313Y	36	cSVD	cSVD, VBD, Proteinuria
31	F	p.Ala143Thr p.Leu394Pro	41	–	–
32	F	p.Ala143Thr p.Leu394Pro	41	Stroke	Stroke
33	M	c.818T > C p.Phe273Ser	62	HCM	HCM, Arrhythmia, Proteinuria, cSVD, GI disorders
34	M	N215S	64	HCM	HCM, Arrhythmia
35	F	c.334C > T p.Arg112Cys	62	Proteinuria	Proteinuria, HCM, Arrhythmia, cSVD, VBD
36	F	c.334C > T p.Arg112Cys	70	Arrhythmia	Arrhythmia, HCM, cSVD, VBD
37	M	c.334C > T p.Arg112Cys	37	Proteinuria	Proteinuria, HCM, cSVD, VBD, Acroparesthesia
38	F	c.334C > T p.Arg112Cys	33	Acroparesthesia	Acroparesthesia
39	M	p.Cys63Tyr	46	Acroparesthesia	Acroparesthesia, HCM, Arrhythmia, CKD, Stroke
40	F	p.Arg118Cys	49	–	–
41	M	p.Arg118Cys	18	HCM	HCM
42	F	c.97G > T p.Asp33Tyr	51	GI disorders	GI disorders, HCM
43	M	c.851T > C p.Met284Thr	34	Proteinuria	Proteinuria, HCM, Arrhythmia, CKD
44	F	c.113delG	6	Acroparesthesia	Acroparesthesia, Proteinuria, TIA
45	F	c.113delG	8	GI disorders	GI disorders, Proteinuria, Acroparesthesia
46	F	c.335G > A Arg112His	22	Proteinuria	Proteinuria
47	F	c.860G > A	8	Acroparesthesia	Acroparesthesia, HCM, Proteinuria, GI disorders
48	F	c.860G > A	49	GI disorders	GI disorders, HCM, Proteinuria
49	F	c.779G > A	56	Proteinuria	Proteinuria, HCM
50	F	c. 846_847delTC	18	Proteinuria	Proteinuria
51	M	c. 846_847delTC	17	Proteinuria	Proteinuria, HCM
52	F	c. 846_847delTC	15	Proteinuria	Proteinuria, PM, TIA
53	M	C172Y	12	GI disorders	GI disorders, Proteinuria
54	F	C172Y	20	Proteinuria	Proteinuria, HCM
55	M	C172Y	23	Proteinuria	Proteinuria
56	M	c. 846_847delTC	62	Proteinuria	Proteinuria
57	M	C172Y	14	GI disorders	GI disorders, HCM, Proteinuria
58	F	c. 846_847delTC	25	Stroke	Stroke, HCM, PM, Proteinuria, TIA
59	F	c. 846_847delTC	12	Acroparesthesia	Acroparesthesia, Proteinuria
60	F	C172Y	5	–	–

*HCM* hypertrophic cardiomyopathy, *cSVD* cerebral small vessel disease, *VBD* vertebrobasilar dolichoectasia, *CKD* chronic kidney disease, *PM* PaceMaker, *GI* gastrointestinal

All the subjects had 10 ml of blood drawn from an antecubital vein in the morning (at fast) for analysis of the advanced oxidation protein products (AOPP), antioxidant ferric-reducing power (FRAP), and total thiol groups.

The results were compared to those obtained from a population of 77 healthy controls (32 men, mean age 61.5 ± 18.2 years), unrelated to the patients, who represent the normative values at the coordinator centre.

AOPP, a marker of oxidative damage to proteins, were assessed according to Witko-Sarsat et al. [11].

AOPP are well-known biomarkers used to evaluate the oxidative modification of proteins, which occurs through myeloperoxidase (MPO) activity of neutrophils. MPO catalyses the production of hypochlorous acid from hydrogen peroxide and chloride ions; hypochlorous acid is capable of oxidizing plasma proteins to generate AOPP. AOPP levels correlated with plasma concentrations of dityrosine and advanced glycation end-products (AGE)-pentosidine as indices of oxygen-mediated protein damage. AOPP measure highly oxidized proteins, especially albumin, and reflect the protein oxidation derived from neutrophils and monocytes [12]. Moreover, it appears that AOPP are integral part of the non-enzymatic antioxidant system of plasma proteome. Oxidized fibrinogen, a molecule responsible for the positivity of AOPP chemical reaction, is bound to apolipoprotein(a), which could compete with plasminogen for its binding sites on fibrinogen, causing inhibition of fibrinolysis and promoting cardiovascular damage. Though it is suggested that the AOPP system functions as a double-edged weapon of the plasma proteome, it by no means diminishes its diagnostic value as an oxidative stress biomarker [13]. For this reason and considering that oxidative modification products of proteins have several advantages such as early formation, longer lifespan, greater stability and reliability in comparison with other oxidative markers [13], we have chosen AOPP as biomarker to test if protein-oxidative changes occur in FD.

In order to measure non-enzymatic anti-oxidant properties, FRAP was assessed in plasma samples, according to Benzie and Strain [14]. FRAP reflects the concentration of low molecular-weight non-enzymatic antioxidants and is independent of the concentration of proteins [15]. We selected FRAP assay for this study because it gives fast and reproducible results, permitting, at the same time, a more objective evaluation of the non-protein antioxidant activity.

The content of plasmatic total thiols (t-SH) was estimated by evaluation of the sulfhydryl groups present in the molecules, following the protocol described by Hu [16]. Sulfhydryl groups (-SH) are responsible for maintaining the structure and function of proteins, enzymes and membranes as well as they can decrease the damage caused by oxidative stress.

Finally, lyso-Gb3 was analysed on dried blood spot (DBS) on different laboratories and data acquired at the coordinator centre for analysis.

We did not perform any statistical analysis between AGAL-A activity in peripheral blood leukocytes and oxidative stress parameters because in our cohort most of the cases are female (66%), and in female AGAL-A activity is not a reliable marker of disease [1].

In a subgroup of eight patients in whom the diagnosis of FD was made being relatives of symptomatic cases, we have monitored oxidative stress biomarkers, Lyso-Gb3 and eventual pre-clinical organ involvement over time. During this observational time, no additional therapies were undertaken, lifestyle did not change, and no unrelated FD diseases occurred.

The Ethic Committees of each centre approved the study, and patients provided informed consent to participate in this study.

### Statistical analysis

Statistical analysis was performed using SPSS Statistics (Statistical Package for Social Science, 20.0 version for Windows). Quantitative data were given as mean ± SD. Normal distribution was determined using Kolmogorov–Smirnov test. For comparisons between two groups, Student’s *t* test and Mann–Whitney *U* test were used as appropriate. Spearman correlation test was performed between the variables. *p* value < 0.05 was considered statistically significant.

## Results

Table 1 shows the demographic, molecular and clinical features of the patients. We have enrolled 60 Italian genetically proven FD subjects (20 men, mean age 46.3 ± 17.3 years, mean disease duration 14.9 years). For the sake of completeness, cases numbers 25 and 30, harbouring the D313Y mutation, and cases 40, 41 (Arg118Cys) remained of no obvious interpretation. In these cases, an extensive work up for other disease explaining organ damage was negative (including NGS screening for genes involved in hypertrophic cardiomyopathy (HCM) in cases 41). Family history of cases 40 and 41 was also positive for renal failure and heart diseases in previous generations.

None of the enrolled patients was smoker or heavy drinker, and no additional diseases were reported. In eight cases (numbers 1, 2, 19, 30, 31, 32, 40, 41 Table 1), at the time of the genetic confirmation, multi organ screening was either negative or revealed a sub-clinical organ impairment.

Hypertrophic cardiomyopathy and arrhythmia were significantly more frequent in male (*p* = 0.002 and *p* = 0.043 respectively) (Table 2). Age of stroke onset was earlier in females than males (37.73 years vs 43.5 years, *p* = 0.046), whereas gastrointestinal involvement was earlier in males (10 years) than in females (27.43 years, *p* = 0.02) (Table 3).

**Table 2:** Differences among sex in clinical features of FD

	Gender		
	M	F	*p*
Hypertrophic cardiomyopathy	14 (70%)	11 (28.2%)	**0.002**
Arrhythmia	8 (42.1%)	7 (17.5%)	**0.043**
Myocardial ischemia	1 (5.3%)		0.322
Proteinuria	14 (70%)	20 (50%)	0.141
Renal failure	2 (10%)	2 (5%)	0.595
Stroke	4 (20%)	15 (37.5%)	0.170
Acroparesthesia	7 (35%)	17 (43.6%)	0.525
Vertebrobasilar dolichoectasia	5 (25%)	7 (17.9%)	0.524
Gastrointestinal involvement	3 (15%)	10 (25.6%)	0.351

*M* male, *F* female, *P* p value

Bold values indicate statistically significant

**Table 3:** Age of onset of different clinical features among FD gender

AGE OF ONSET years (standard deviation)			
	Male	Female	*p*
Hypertrophic cardiomyopathy	44.36 (13.62)	52.38 (9.46)	0.17
Arrhythmia	55.71 (13.27)	58.67 (14.74)	0.762
Myocardial ischemia	62 (Only one case)		
Proteinuria	41.67 (15.33)	39.13 (14.81)	0.693
Renal failure	47 Only one case		
Stroke	43.5 (0.71)	37.73 (3.49)	0.046
Acroparesthesia	18.8 (11.78)	26.2 (13.79)	0.298
Vertebrobasilar dolichoectasia	49.5 (17.68)	49.25 (21.28)	0.989
Gastrointestinal involvement	10	27.43 (17.51)	0.02

**Fig. 1:**
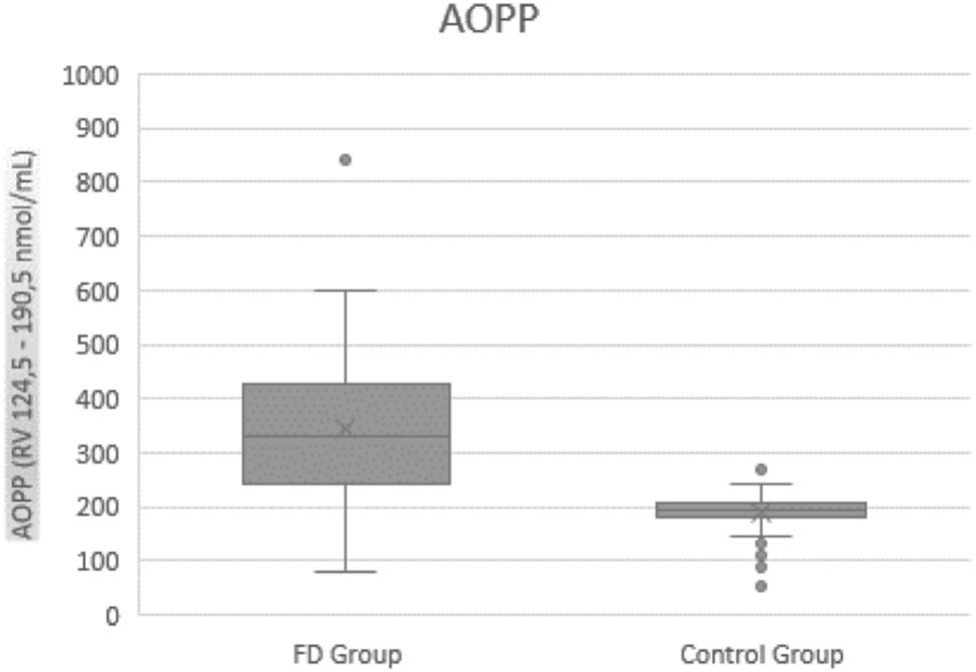
AOPP values in FD patients and controls

**Fig. 2:**
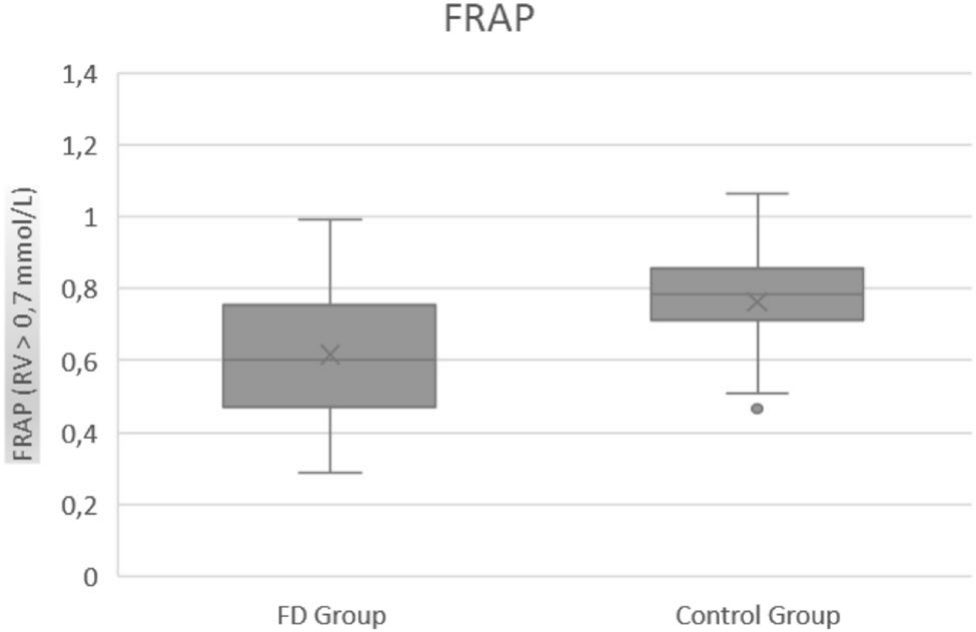
FRAP values in FD patients and controls

**Fig. 3:**
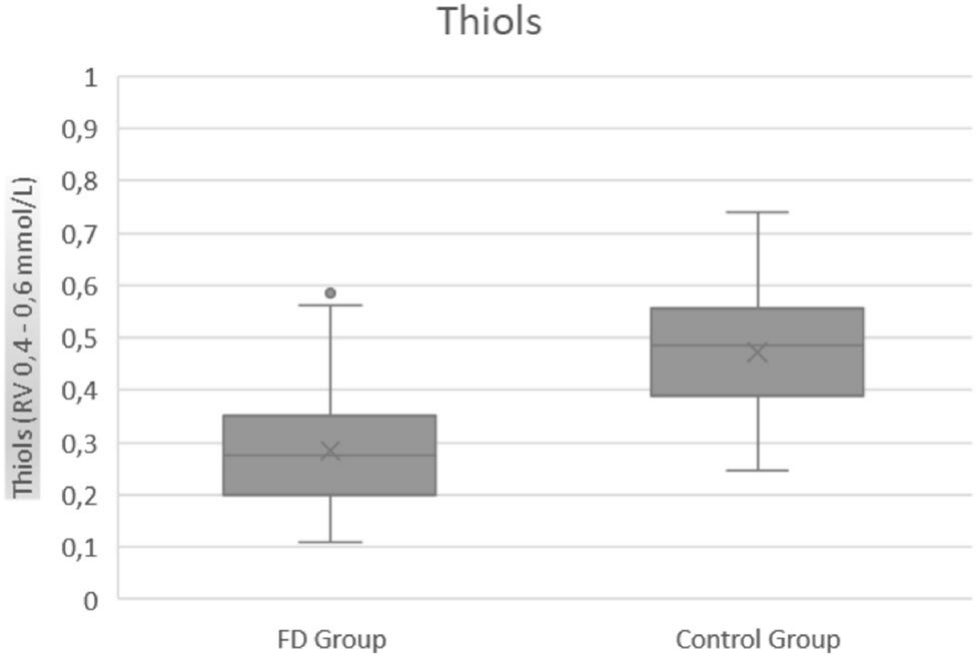
Thiols values in FD patients and controls

Table 4 presents values of oxidative stress parameters among patients and controls. As expected, the average values of all oxidative stress parameters in healthy controls were in the normal range, while AOPP levels were significantly higher (*p* < 0.00001, Fig. 1) and FRAP (Fig. 2) and thiols (Fig. 3) significantly lower in FD than healthy controls (*p* < 0.00001). These parameters were not significantly different neither between enzyme replacement therapy (ERT) or non-ERT patients (AOPP: *p* = 0.129, FRAP *p* = 0.441, thiols *p* = 0.315) or gender (*p* = 0.799 for AOPP, *p* = 0.901 for FRAP, *p* = 0.367 for thiols respectively), or age of onset of the disease.

**Table 4:** Values of oxidative stress (AOPP, FRAP, Thiols) among patients and controls. RV: reference values. IQR: interquartile range

	Median Value (IQR) Patients	Median Value (IQR) Controls	*P*
AOPP (RV 124.5–190.5 nmol/mL)	342.74 (146.15)	185.32 (43.93)	**<** ***0.000001***
FRAP (RV > 0.7 mmol/L)	0.61 (0.18)	0.78 (0.12)	**<** ***0.000001***
Thiols (RV 0.4–0.6 mmol/L)	0.28 (0.10)	0.47 (0.12)	**<** ***0.000001***

Bold values indicate statistically significant

No association was observed between hypertrophic cardiomyopathy, arrhythmia, myocardial ischemia, proteinuria, renal failure, acroparesthesia, vertebrobasilar dolichoectasia, gastrointestinal involvement and oxidative stress parameters. The only observed association was between FRAP levels and stroke (*p* = 0.02), with pathologic levels of FRAP inversely associated with stroke.

No correlation between oxidative stress parameters and overall time of FD was found (AOPP *p* = 0.324; FRAP *p* = 0.192; thiols *p* = 0.581). Moreover, no correlation was observed between Lyso-Gb3 levels and AOPP (*p* = 0.415), FRAP (*p* = 0.51) or thiols (*p* = 0.827).

Comparing the classical (onset under 50 years of onset) and late onset FD forms, significant higher values of AOPP levels were observed in the late onset form (*p* = 0.04, Table 5). The same was observed in our control group (< 50 years old vs > 50 years old, Table 6). Comparing late FD forms with the control subgroup over 50 years, a significant difference in the average values of AOPP, FRAP and thiols was found, as shown in Table 7.

**Table 5:** Average values of oxidative stress parameters in classic vs late onset FD

	*N*	Mean	Standard Deviation	*p*
AOPP				
Late	10	428.5900	187.40270	**0.040**
Classic	48	324.8558	131.47583	
FRAP				
Late	8	0.62338	0.139265	0.903
Classic	46	0.61476	0.189121	
Thiols				
Late	10	0.24570	0.079635	0.227
Classic	48	0.29217	0.114189	

Bold value indicates statistically significant

**Table 6:** Average values of oxidative stress parameters in healthy controls (< 50 ys old vs > 50 ys old)

	AGE	*N*	Mean	Std. Deviation	Std. Error Mean	*p*
AOPP	≥ 50	58	195.7238	33.88970	4.44994	**0.007**
	< 50	19	164.9105	61.68450	14.15139	
FRAP	≥ 50	58	0.78448	0.133982	0.017593	**0.014**
	< 50	19	0.69931	0.104409	0.023953	
Thiols	≥ 50	58	0.48364	0.129588	0.017016	0.154
	< 50	19	0.43768	0.086380	0.019817	

Bold values indicate statistically significant

**Table 7:** Average values of oxidative stress parameters in late onset FD vs healthy controls (> 50 years old)

	*N*	Mean	Std. Deviation	Std. Error Mean	*p*
AOPP					
Late Form	10	428.5900	187.40270	59.26194	**< 0.000001**
Controls ≥ 50	58	195.7238	33.88970	4.44994	
FRAP					
Late Form	8	0.62338	0.139265	0.049238	**0.002309**
Controls ≥ 50	58	0.78448	0.133982	0.017593	
Thiols					
Late Form	10	0.24570	0.079635	0.025183	**< 0.000001**
Controls ≥ 50	58	0.48364	0.129588	0.017016	

Bold values indicate statistically significant

Given the difference on age described above, we performed a bivariate analysis, including age, and disease overall time (defined as difference between actual age and age at first disease manifestation) and cardiomyopathy as independent variables; the difference was significant despite correction for age (*p* = 0.047) and disease overall time (*p* = 0.045).

**Fig. 4:**
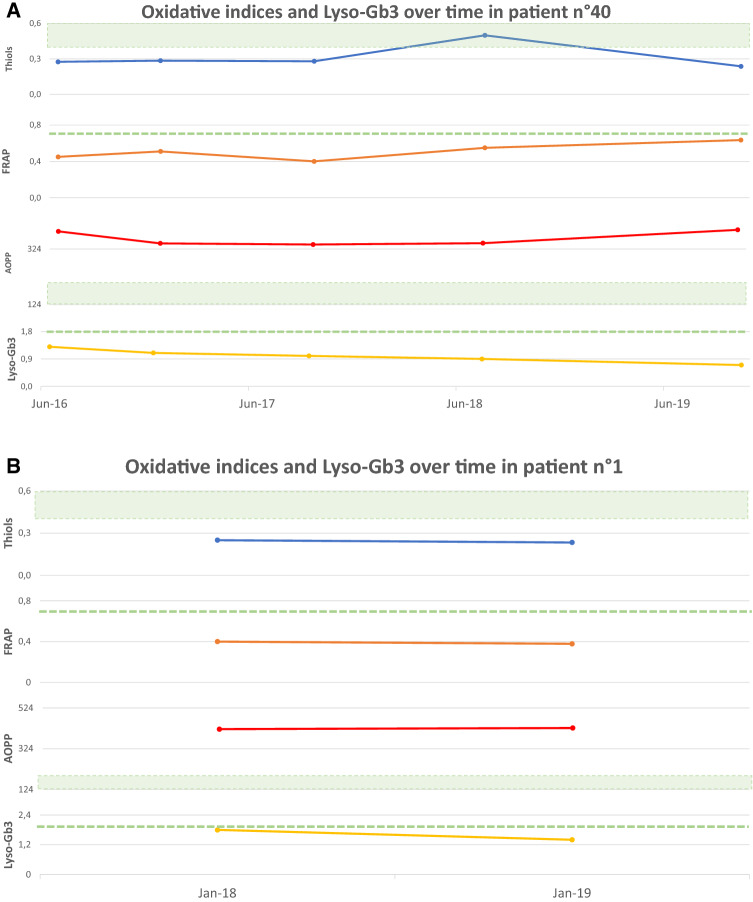

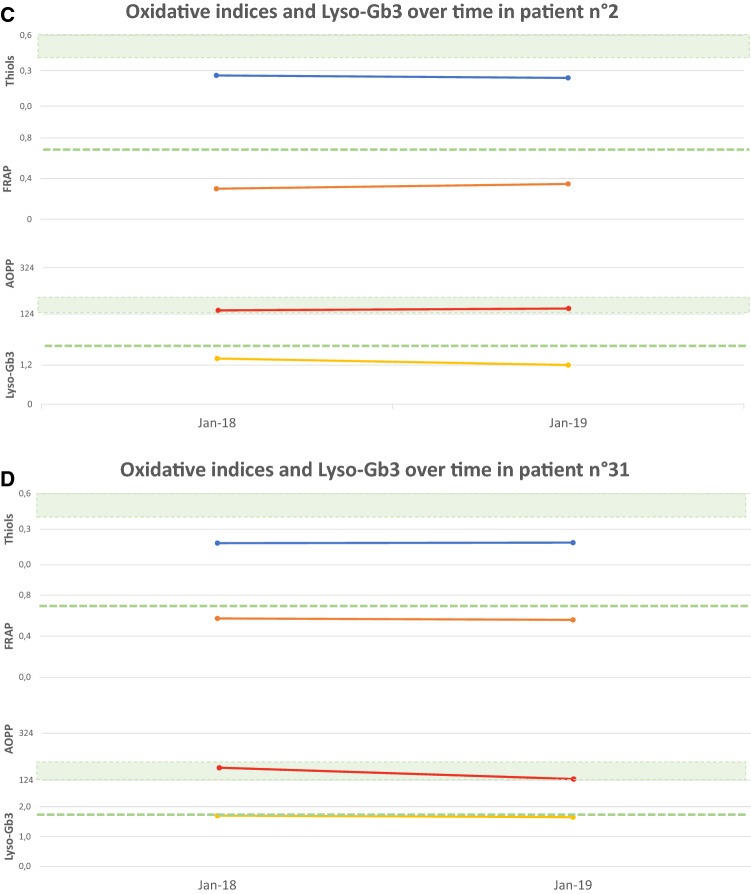

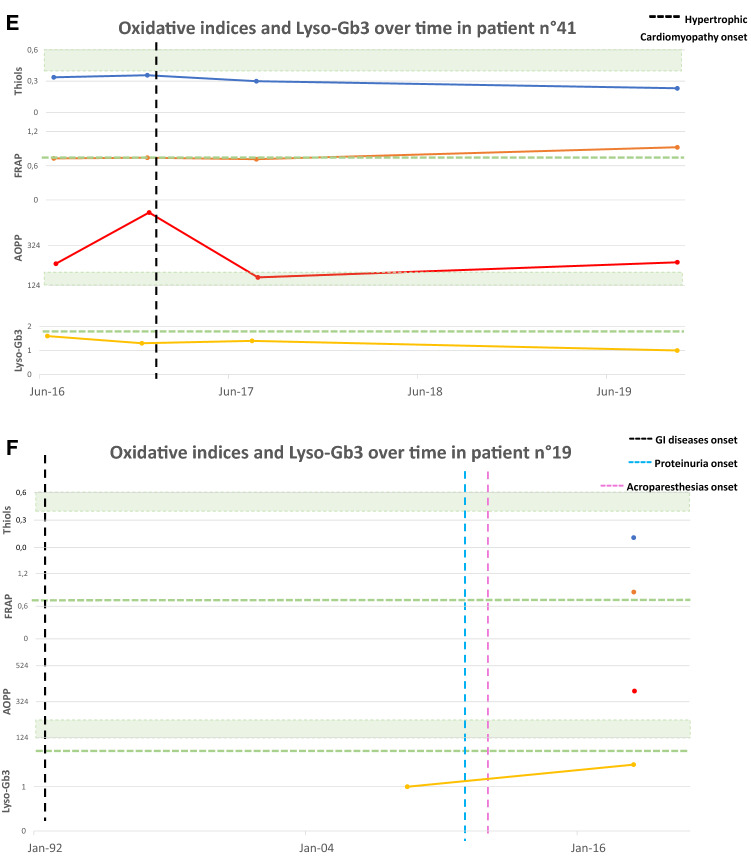

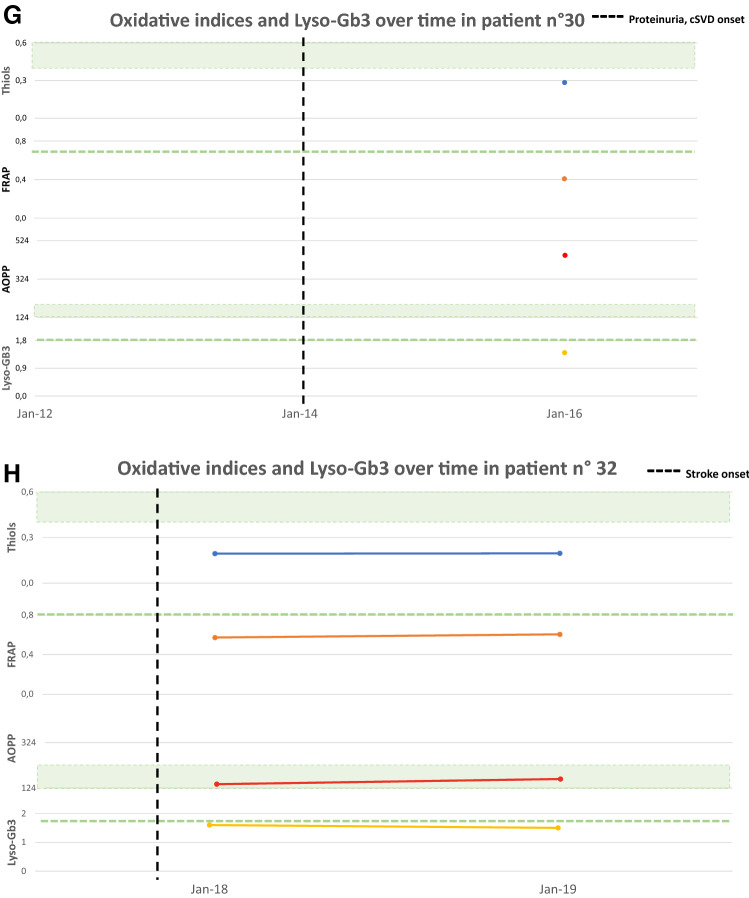
Oxidative stress parameters (blue: Thiols, orange: FRAP, red: AOPP) and Lyso-Gb3 (yellow) values over time. The green area and dashed lines show reference values (RV) (AOPP < 190.5 nmol/mL; Lyso-Gb3 < 1.8 ng/mL; Thiols > 0,4 mmol/L; FRAP > 0.7 mmol/L). RV are obtained from a population of 77 healthy controls (32 men, mean age 61.5 ± 18.2 years), unrelated to the patients, recruited at the coordinator centre. The vertical dashed line indicates the appearance of a clinical event related to FD natural history

Figure 4 shows oxidative stress parameters over time in a subgroup of eight treatment-naïve patients all with normal Lyso-Gb3 (reference values < 1.8 ng/mL) at the enrolment time, at the follow up and the relation with significant clinical or subclinical events. Four patients (4A, B, C and D) showed a significant alteration in oxidative stress parameters already before the appearance of organ impairment or signs of FD features at the last follow up. Other two patients (4E and 4F) showed pathological oxidative stress parameters already before the appearance of organ damage, while Lyso-Gb3 remained normal. The last two cases (4G and 4H) showed pathological oxidative stress parameters and normal Lyso-Gb3 at the time of the diagnosis, having already presented impairment of one or more organ target.

## Discussion

In this study, we observe a significant imbalance of the oxidative stress status in FD patients in different disease stage, including those cases in which Lyso-Gb3 was normal and\or organ damage was not yet manifested.

ERT has changed the natural history of the disease in FD patients. Initiation of ERT in childhood could slow, or even stop the progression of organ damage before irreversible changes occur [17]. Although the exact pathogenesis of FD is still under unclear, the accumulation of Gb3 and lyso-Gb3 in vascular endothelia and subsequent inflammation is thought to play a crucial role, whereas early ERT initiation may reduce lyso-Gb3 deposition and mitigate disease progression [18]. ERT seems to be less effective in patients with both delayed diagnosis and initiation of ERT, and in classic FD there is a tendency to initiate therapy when early symptoms or signs of organ damage occurs [18]. Moreover, in a recent consensus recommendation paper [17] it has been suggested that asymptomatic boys may benefit from an earlier initiation of ERT based on the following criteria: presence of a pathogenic GLA variant responsible for the classic phenotype, family history of disease severity in males, undetectable AGAL‐A activity in peripheral blood leukocytes and plasma lyso-Gb3 over 20 nmol/L. Therefore, biomarkers could be used as reliable features to guide the clinician in the therapeutic management.

On the other hand, in recent years there are several evidences that the accumulation of Lyso-Gb3 is not the only mechanism underlying the progression in FD. Several authors have put forward the hypothesis that oxidative stress has a key role in the progression of organ damage in FD. In 2012, Biancini and collaborators demonstrated that in FD pro-oxidant states occur and seem to be induced by Lyso-Gb3 [19]. In their study, decreased levels of antioxidant defences (reduced glutathione, glutathione peroxidase activity and increased superoxide dismutase/catalase ratio in erythrocytes), and high levels of malondialdehyde (MDA) and protein carbonyl groups products (product of proteins and lipid damage) were observed [19]. Interestingly, urinary Gb3 levels were positively correlated with the plasma levels of IL-6, carbonyl groups and MDA, suggesting a possible link between lyso-GB3 and oxidative stress [19]. In 2016, we have also hypothesised that mitochondrial DNA haplogroups may be involved in modulating the oxidative stress in FD, thus explaining the heterogenous expression of FD phenotype [20].

In 2015, Chimenti et al. have evaluated the role of oxidative stress in FD cardiomyocyte dysfunction; they found that GB3-induced myocardial ROS production provides a major contribution to cardiac dysfunction of FD cardiomyopathy [21].

Recently, Ravarotto et al. have documented oxidative stress activation and oxidative stress signalling (higher p22phox expression and MDA levels), suggesting a possible role of oxidative stress in cardiovascular-renal remodelling pathophysiology [22]. In their work, patients were all in ERT, suggesting that oxidative stress occurs despite ERT in FD related-left ventricular hypertrophy. Similar conclusions were reached by Biancini et al. in 2016 that showed higher lipid peroxidation levels in FD before ERT, which could not be reversed by the treatment [7]. These findings have led to suppose a role for oxidative stress-targeted therapies in addition to ERT in FD.

It has been shown that Gb3 accumulation is not limited to the lysosomes but also in endothelial cells membranes, inducing deregulating endothelial NO synthase activity with reduced NO production that favours oxidative stress [23].

Our results confirm oxidative stress activation in FD; in particular, we have observed high levels of AOPP and reduced levels of antioxidants (FRAP and thiols) in patients compared to healthy controls; these parameters were altered despite ERT, confirming Biancini observation [7]. Given the others study on oxidative stress published up so far, we might assume a vicious cycle between Lyso-Gb3 deposition and oxidative stress: Lyso-Gb3 deposition induces organ damage, endothelial dysfunction, increases vascular superoxide and free radical formation, oxidative DNA damage and altered oxidative response, which in turn enhances organ damage and remodelling.

We have not observed an association between oxidative stress parameters alteration and disease manifestations. The inverse association between pathological values of FRAP and stroke (the opposite of what one would expect, given the possible role of oxidative stress in cerebral small vessels disease) is unclear and requires further investigation.

We also found significantly higher levels of AOPP between late onset and classic FD, despite correction for age and disease overall time.

Interestingly, in a group of eight treatment-naïve subjects/patients with FD-related mutations and normal Lyso-Gb3 levels we have found pathological oxidative stress parameters. Lyso-Gb3 levels didn’t correlate with the evaluated biomarkers; we can assume that these parameters reflect early activation of oxidative stress and organ damage, before lyso-Gb3 elevation, but further studies are needed. This group consisted of some challenging cases: two pre-symptomatic young females with the pathogenic p.Phe113Leu, traditionally related to a late-onset phenotype, in which lysoGB3 levels could be sometimes normal [24]; a 10-year-old male with a classical phenotype and normal levels of LysoGB3; two females with the recently considered “likely benign” variant D313Y but manifesting some clinical manifestations of FD [25, 26]; a 18-year-old male with HCM, and his asymptomatic mother, with the Arg118Cys variant with conflicting interpretations of pathogenicity​ according to ClinVar [27–29]. These cases rise some queries about the diagnostic value of lyso-GB3 in some settings, and confirms that diagnosis of FD cannot be made solely on genetic basis. Histopathologic studies are crucial in challenging cases to confirm the diagnosis of FD but may be invasive (as in myocardial biopsy), and not always available in some settings. Unfortunately, we do not have histopathological studies in our cases. The altered parameters of oxidative stress in the above cases may suggest an early involvement of redox imbalance in FD-induced organ damage, disease onset and progression. Although some authors have demonstrated the progression of organ damage in ERT patients, the early treatment is considered to be the key to dramatically change the natural history of disease in these patients [18, 30, 31]. Our results are not enough to state that ERT should be started in subjects with FD-related mutations who do not have signs or symptoms of disease but signs of oxidative stress, however they pave the way for further studies. To date, beside AGAL-A activity in males, the only surrogate biomarker recognized in FD is Lyso-Gb3. Considering what we have observed, specific oxidative stress markers could be even more sensitive than Lyso-Gb3 in the early phase of the disease, thus representing new early biomarkers with a potential role in helping diagnosis and time to treatment decision.

Our study has some limitations. We have enrolled patients at different stage of disease, and the timing of the biomarkers analysis was variable from patient to patient. Moreover, most of our cases are females, thus we could not correlate AGAL-A activity with the other findings. No complete data about all possible comorbidities were available, although patients didn’t have significant comorbidities such as cancer or other inflammatory disorders.

In conclusion, oxidative stress occurs in FD in both treated and naïve patients, highlighting the need of further collaborative research in oxidative stress-targeted therapies in addition to ERT in order to further slow disease progression. Furthermore, oxidative stress biomarkers may represent early markers of disease in treatment-naïve patients with a potential role in helping interpretation of FD-related mutations and time to treatment decision.
